# The Impact of Green Practices in Value Chain on Firm Performance in the Context of a Developing Country

**DOI:** 10.12688/f1000research.73589.2

**Published:** 2022-06-07

**Authors:** Jeen Wei Ong, Gerald Guan Gan Goh, Sally Hui Siang Yong

**Affiliations:** 1 Faculty of Management, Multimedia University, Cyberjaya, Selangor, 63100, Malaysia; 2 Faculty of Business, Multimedia University, Ayer Keroh, Melaka, 75450, Malaysia; 3 Power Logic (M) Sdn. Bhd., Puchong, Selangor, 47100, Malaysia

**Keywords:** Green Practices, Value Chain, Firm Performance, Malaysian Corporations, Multilinear Regression Analysis

## Abstract

**Background:** Companies need to go green to remain relevant. Previous studies have confirmed that going green leads to superior performance for companies. However, research of green practices in a value chain requires further attention, especially in identifying the green value chain activities that lead to superior performance. A value chain analysis focuses on identifying competitive advantages of firms through five primary and four support activities.

**Methods:** This study extends from Ong et al. (2019), who developed and validated the instrument for the nine green value chain activities, to also examine their effect on firm performance. The 207 valid responses in this study are collected through a questionnaire survey of the sampling frame consisting of companies in Bursa Malaysia and the Federation of Malaysian Manufacturers Directory.

**Results:** The findings reveal that the companies’ green practices in primary value chain activities are higher than in the supporting value chain activities. Technological development is the activity with the lowest green attention among the nine value chain activities. Our multiple regression analysis shows that 25% of the variation in firm performance can be significantly explained by the nine green value chain activities. In terms of the individual green value chain activities, green technology development is the only activity that can positively and significantly explain firm performance.

**Conclusions:** The findings of the study suggest that companies intending to build their green core competence need to engage in green technology development. Companies that go green for the purpose of complying to regulations and fulfilling minimum customers’ demands can still embed green practices into their green value chain without compromising their performance.

## Introduction

It is no longer a choice, but it is instead becoming necessary for companies to be environmentally friendly or greener in their business operations. Consumers, especially millennials, show a preference for environmentally friendly companies and products. ^1^ Companies need to embed an environmentally friendly approach completely into multiple dimensions of their business operations in order capture the value created in the form of performance. ^1^ Various research findings have confirmed that green practices in businesses lead to greater performance (e.g., Refs. 2– 4). However, there is still an opportunity to further study green practices from the value chain perspective. The value chain, introduced by Porter, ^5^ provides a comprehensive analysis of the value creation activities within a company. Anchoring green research to businesses from the value chain perspective can provide further detail on the actual value creation activities that lead to superior performance.

In analysing the competitive advantage of a firm, Porter ^5^ introduced value chain analysis to distill the value creation activities within a company into five primary and four support activities. The five primary activities are the inbound logistics, operations, outbound logistics, marketing, and sales and services, denoting the complete value creation process from materials to after-sales services to the customers. ^5^ The procurement, firm infrastructure, technology development, and human resources management support the primary activities as the support activities in the value creation process. ^5^ The concept of green value chain is to embed the green elements into the value chain activities of the firms. Studies by Handfield, Walton, Seegar, et al. ^6^ and Hartman and Stafford ^7^ are among the early researches that explore the idea of a green value chain. Subsequently, Ndubisi and Nair, ^8^ Yong, Goh and Ong, ^9^ Anthony Jnr ^10^ and Ong et al. ^11^ are among the studies that consider the full value chain in the context of Porter. ^5^ The studies by Hartman and Stafford, ^7^ Ndubisi and Nair, ^8^ Yong et al., ^9^ are conceptual in nature without empirical evidence that green practices benefit the firm performance. Handfield, Walton, Seegar, et al. ^6^ on the other hand focused on examining the green practices of five companies in furniture industry. Both Anthony Jnr ^10^ and Ong et al. ^11^ have operationalized the value chain activities but neither of these studies examined the impact of the green value chain activities on firm performance. Anthony Jnr ^10^ studies the impact of green value chain activities on the sustainable value chain practices while Ong et al. ^11^ focus on validating the instrument. Thus, empirical evidence to support the impact of green value chain on firm performance is insufficient based on existing studies.

## Methods

**Figure 1.**
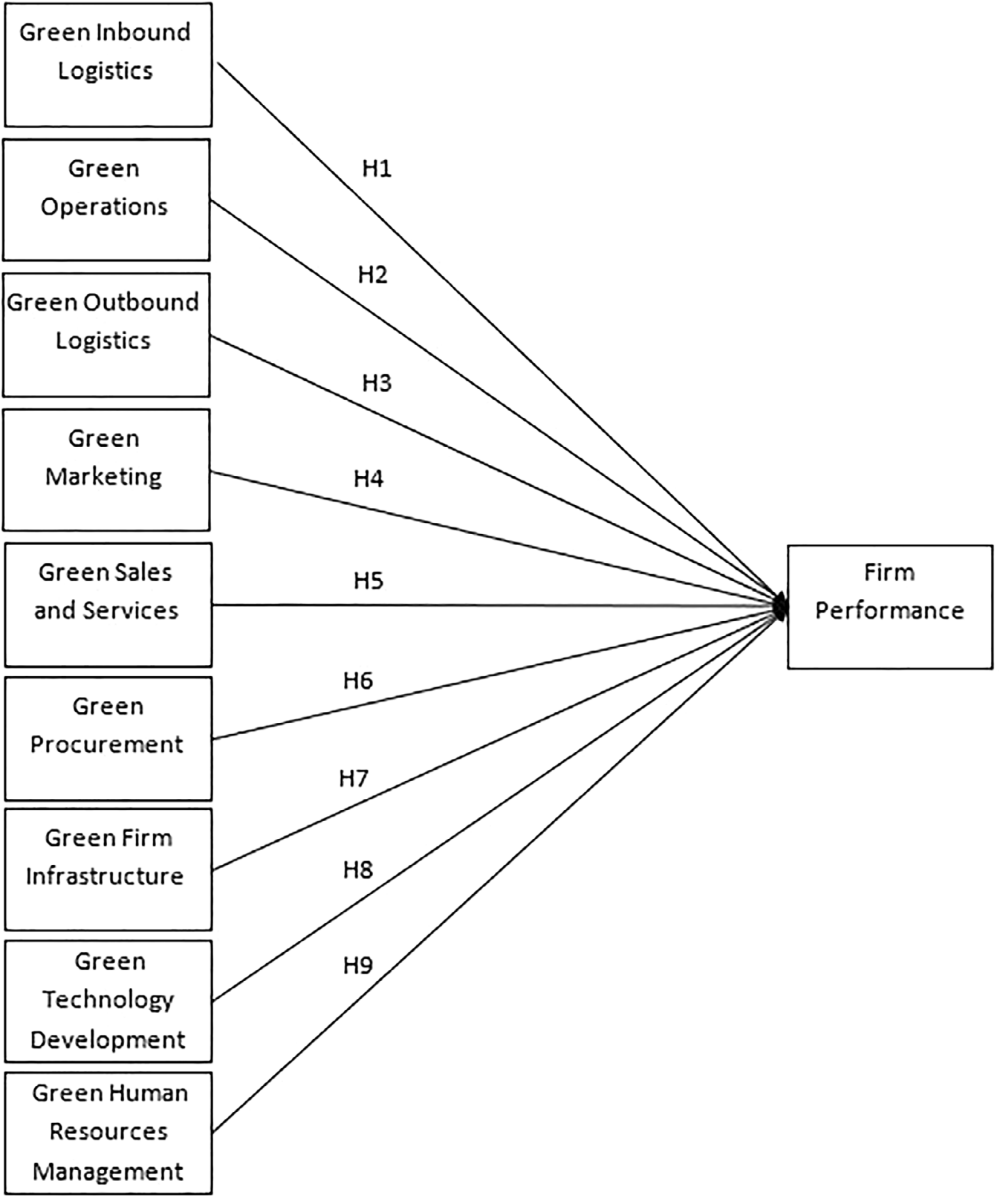
Research Framework. Based on the research framework, the following hypotheses are developed. H1: The green inbound logistics has a significant positive impact on firm performance. H2: The green operations has a significant positive impact on firm performance. H3: The green outbound logistics has a significant positive impact on firm performance. H4: The green marketing has a significant positive impact on firm performance. H5: The green sales and services has a significant positive impact on firm performance. H6: The green procurement has a significant positive impact on firm performance. H7: The green firm infrastructure has a significant positive impact on firm performance. H8: The green technology development has a significant positive impact on firm performance. H9: The green human resources development has a significant positive impact on firm performance.

This study aims to examine the impact of the green value chain activities on firm performance. The research framework is presented in Figure 1, followed by the hypotheses developed for the study.

The survey instrument for the nine activities of the green value chain was developed using responses from semi-structured interviews with 35 companies across different business sectors. These companies were selected based on their significant involvement in championing the green initiatives in their operations. The interviewees from these companies were in managerial positions from various business functions. We asked the respondents to describe the company and their experience with the company to verify their understanding of the company. In capturing the green practices in the company value chain, the respondents were asked to list the important green activities under each of the nine value chain activities suggested by Porter. ^5^ For instance, the interviewees were asked to list and describe all the green inbound activities performed by the companies. This was repeated for all the other nine value chain activities. The data collected from all the 35 companies were then compiled to identify the key themes for each green value chain activities. These key themes were then compiled as the questionnaire items. There are a total of 99 items for the nine green value chain activities. On the other hand, the instrument to measure the firm performance is adopted from Ong ^12^ and consists of seven items. All the items are measured using the seven-point itemized rating scale with one indicating strongly disagree and seven indicating strongly agree.

The survey form also consisted of a cover letter and an informed consent statement. These documents communicated the purpose of the study, and detailed the research sponsor and researchers, the research procedure, the voluntary nature of the study, the possible risks and benefits of participating in the research, and the confidentiality of the respondents’ identity. The respondents were informed that by returning the survey form, they indicated their consent to participate in the research but that they could withdraw their participation by informing the researchers.

A census method was used to contact all 1,150 companies listed in the main market and ACE market of Bursa Malaysia and the Federation of Malaysian Manufacturers Directory with complete mailing information. Letters were sent out to all. Fourteen letters were returned due to inaccurate mailing information. By the end of the survey, 207 valid responses were received. The assurance of confidentiality of the identity of respondents and the absence of fixed or expected responses in the survey aimed to ease the possible issue of common method variance. ^13^

The data collected was analysed using SPSS version 26 ( IBM SPSS Statistics, RRID:SCR_019096). Alternatively, GNU PSPP is a free open-source software that can be used to perform similar functions. The results are presented in the next section, starting with the presentation of a brief profile of the responding companies, followed by the reliability and validity analysis and mean analysis for all the variables. Lastly, the impact of the nine green value chain activities on firm performance is tested using multiple linear regression analysis.

## Results

The demographic profile of the companies, in terms of the company size, years of operation, and status of ownership, are presented in Table 1. The statistical results show that more than half of the companies have 1,000 or fewer employees. In terms of years of operation, close to half of them have operated for 10 years and lesser. A vast majority of them are locally owned.

**Table 1.** Profile of responding companies.

Variable	Attribute	Frequency	Percentage
Company size (No of employees)	500 and lesser	52	25.10
	501 to 1,000	65	31.40
	1,001 to 1,500	35	16.90
	1,500 to 2,000	21	10.10
	2,001 and more	34	16.40
Years of operation	10 years and below	91	44.00
	11 to 20 years	72	34.80
	21 to 30 years	22	10.60
	31 years and above	22	10.60
Status of ownership	Local	173	83.60
	Foreign	34	16.40

We performed exploratory factor analysis on the all the items for the nine green value chain activities and the firm performance. Results show that the Kaiser-Meyer-Olkin (KMO) is higher than 0.80 and the Bartlett’s test of sphericity is significant at the 95% confidence level. Two items, one each for Operations and Services were removed due to cross loading. The rule of thumb used was that the loading must be above 0.40 in one factor only. ^14^ The inter-item consistency for all individual variables was tested using Cronbach’s Alpha, with results indicating a satisfactory level of inter-item consistency for all variables. The details are presented in Table 2.

**Table 2.** The validity, reliability and mean analysis.

Variable	# of items	Cronbach’s alpha	Mean	Standard deviation
Inbound Logistics	15	0.958	4.67	1.26
Operations	16	0.974	5.00	1.02
Outbound Logistics	9	0.968	5.23	1.00
Marketing and Sales	10	0.966	5.26	1.11
Services	11	0.941	5.66	1.03
Procurement	7	0.966	5.00	0.85
Technology Development	9	0.974	3.68	1.44
Human Resource Management	10	0.952	4.00	1.12
Corporate Infrastructure	10	0.960	4.66	0.93
Firm Performance	7	0.941	4.91	0.96

In addition, Table 2 presents the mean and standard deviation for each variable. The mean for the nine green value chain activities ranged from 3.68 to 5.66 on the seven-point scale. The activity with the highest mean score was services, with a mean of 5.66 and a standard deviation of 1.03. On the other hand, technology development had the lowest mean with a score of 3.68 and a standard deviation of 1.44. Firm performance had a mean score of 4.91 and standard deviation of 0.96 on the seven-point scale.

Table 3 shows the results of the multiple linear regression analysis. This analysis was used to test the effect of the nine green value chain activities on firm performance. The correlation coefficient, R, was 0.500 and the R-squared was 0.250 (F = 7.282; p < 0.05). This shows that 25% of variation in the firm performance is explained by the nine green value chain activities. Among the nine green value chain activities, only technology development was found to have a significant impact on firm performance (Beta = 0.257; t-value = 2.815; p < 0.05). Thus, only hypothesis 8 (H8) is not rejected. The green technology development activities is found to have significant positive impact on firm performance.

**Table 3.** Multiple linear regression analysis for effect of green value chain activities on firm performance.

**R**	0.500
**R Square**	0.250
**F**	7.282
**Sign**	0.000

Variables	Beta	t-value	Sig.
Inbound Logistics	0.109	1.365	0.174
Operations	-0.036	-0.385	0.701
Outbound Logistics	0.040	0.481	0.631
Marketing and Sales	-0.048	-0.561	0.576
Services	0.077	1.023	0.308
Procurement	-0.027	-0.345	0.730
Technology Development	0.257	2.815	0.005
Human Resource Management	0.109	1.215	0.226
Corporate Infrastructure	0.156	1.727	0.086

## Discussion

The general profile of the sample companies in the study is relatively small and young with more than half of them having employee numbers below 1,000 and near to half of them having been established for less than 10 years. In terms of the value chain activities, these companies embed green practices into the primary value chain better than the support activities, except for the inbound logistics. The smaller size of suppliers could contribute to lesser enforcement of green practices in this primary value chain activity. It is also observed that the green practices in the four support activities are low. Without the right technology, infrastructure, procurement processes, and human resources, the effectiveness and sustainability of the green practices in the primary activities remain questionable.

Similar to previous studies, ^2^^–^^4^ our study found that going green can enhance firm performance. In the context of green value chain, our findings suggest that the involvement of companies in the research and development of green technologies is crucial for companies to gain superior performance. This signifies the importance of proprietary green technology to firm performance. The visibility of a companies’ involvement in green technology development could be a contributor, especially for those involved in the business-to-business sector. The capability and experience in green proprietary technology development is vital to gain superior performance from going green. However, it is also noted from the findings that the technology development has the lowest score green activities among all nine value chain activities. The findings of our study are not consistent with Anthony Jr’s ^10^ study. He found that all the green value chain activities are significant predictors to the triple bottom line of the firms. This shows the criteria in achieving the broader triple bottom line performance and the narrower economic performance is different in the context of value chain.

In the case of Malaysia, based on this study, the lack of involvement in green technology development is observed. This would require attention from the companies and the policy makers. On the other hand, there is no evidence to show that involvement in green practices inversely affects firm performance. Companies have to make a strategic decision in going green. They could decide to take a strategic move to create core competency by developing the green technology to gain superior performance. The policy makers in encouraging the companies to go green need to ensure the companies involved in green technology development if any incentives were to be granted. On the other hand, companies can decide to use the compliance model by fulfilling the minimum regulatory or customer requirements in going green. They can build their core competency elsewhere. There is no evidence from this study that the latter model could cost their performance. The findings from the green practices in primary and support value chain activities, show that most of the companies could be using the latter model.

## Conclusions

The study extends from Ong et al. ^11^ to further analyse the impact of green value chain activities on firm performance. Our findings support the notion that companies involved in green activities can gain superior performance. There is no evidence suggesting that embedding green practices in the value chain can have a negative impact on firm performance. Companies need to have the correct business model and strategy in approaching the trend of increasing demand to be environmentally friendly. Nonetheless, future research could further validate the instruments used in this study. Future research could also study the existence of mediators or moderators that cause the insignificance of all the primary green value chain activities in explaining the superior firm performance.

## Data availability

Figshare: https://doi.org/10.6084/m9.figshare.14883240.v1 ^15^

This project contains the following underlying data:

• GVC_Dataset_Share.sav (The dataset was collected through a questionnaire)

Data are available under the terms of the Creative Commons Attribution 4.0 International license (CC-BY 4.0).

## Ethical approval and consent

The consent form was attached to the survey form and sent to the respondents as a letter. The form included the research purpose, research sponsor, research procedures, a statement of voluntary participation, confidentiality of the respondents and the company's identity, risks and benefits of participating in the study and a statement on consent agreement. The consent agreement clearly spelled out that completing and returning the completed survey form indicated the respondent's consent to participate in the research.

Ethical approval was granted by the Research Ethics Committee (REC) of Multimedia University. The committee granted the approval after reviewing the self-declared form submitted by the researchers.

The approval number is EA2312021.

## Author contributions

Ong, J. W. involves in data collection and data analysis and completing the first draft of write-up for this article.

Goh, G. G. G. contributes in the items development and editing the final version of this article.

Yong, H. S. S. involves in early stage interviews that subsequently leads to the measurement items development. She assists also in data collection.
