# Supplementary material for: The Impact of Green Practices in Value Chain on Firm Performance in the Context of a Developing Country
Source: F1000Res. 2022 Jun 7;11:264. Originally published 2022 Mar 2. [Version 2] doi: 10.12688/f1000research.73589.2 (PMC9379330; doi:10.12688/f1000research.73589.2)
Supplement: Peer review history

# Peer review: Reviewer response for version 2

**DOI:** 10.5256/f1000research.134448.r139932

**Published:** 2022-08-15

**Authors:** Uchenna Cyril Eze

**Affiliations:** 1 Division of Business and Management, BNU-HKBU United International College, Zhuhai, China

The revisions are appropriate and sufficient. I, therefore, approve the submission without hesitation.

Is the work clearly and accurately presented and does it cite the current literature?

Yes

If applicable, is the statistical analysis and its interpretation appropriate?

Yes

Are all the source data underlying the results available to ensure full reproducibility?

Yes

Is the study design appropriate and is the work technically sound?

Yes

Are the conclusions drawn adequately supported by the results?

Yes

Are sufficient details of methods and analysis provided to allow replication by others?

Partly

Reviewer Expertise:

Mindful consumption, e-business, knowledge management, sustainability in business, green marketing, strategy

I confirm that I have read this submission and believe that I have an appropriate level of expertise to confirm that it is of an acceptable scientific standard.

# Peer review: Reviewer response for version 2

**DOI:** 10.5256/f1000research.134448.r139933

**Published:** 2022-06-28

**Authors:** Sumesh Nair

**Affiliations:** 1 Australian Institute of Business (AIB), Adelaide, SA, Australia

I am fine with the changes made in the article and wish the authors of the paper good luck.

 Best regards,

 Sumesh

Is the work clearly and accurately presented and does it cite the current literature?

Yes

If applicable, is the statistical analysis and its interpretation appropriate?

Yes

Are all the source data underlying the results available to ensure full reproducibility?

Yes

Is the study design appropriate and is the work technically sound?

Partly

Are the conclusions drawn adequately supported by the results?

Partly

Are sufficient details of methods and analysis provided to allow replication by others?

Partly

Reviewer Expertise:

Green/environmental marketing and Ethical Marketing.

I confirm that I have read this submission and believe that I have an appropriate level of expertise to confirm that it is of an acceptable scientific standard.

# Peer review: Reviewer response for version 1

**DOI:** 10.5256/f1000research.77250.r126017

**Published:** 2022-04-11

**Authors:** Sumesh Nair

**Affiliations:** 1 Australian Institute of Business (AIB), Adelaide, SA, Australia

"The Impact of Green Practices in Value Chain on Firm Performance in the Context of a Developing Country" is an interesting read. The introduction portrays the literature gap rather briefly. A brief literature review section would have benefited the paper in terms of elaborating the extant literature's contributions and identifying the gaps in the literature. 

 The methods section would have been a little more detailed with some discussion around the semi-structured interviews. What questions were asked, and how did these responses contribute to the construction of the survey instrument? I am wondering why no hypotheses were used in the study. 

 In a few instances of the discussion section, the researchers used the word 'worrying', it is not clear why some findings worry the researchers. The use of a more neutral language would be ideal in these instances. It would have been great to discuss the implications of the study findings in a separate section of the paper. 

 Overall, the paper presents a very interesting analysis and findings but minimally. I am not sure this is due to any word count requirements of the journal. 

 I wish the authors good luck with this publication.

Is the work clearly and accurately presented and does it cite the current literature?

Yes

If applicable, is the statistical analysis and its interpretation appropriate?

Yes

Are all the source data underlying the results available to ensure full reproducibility?

Yes

Is the study design appropriate and is the work technically sound?

Partly

Are the conclusions drawn adequately supported by the results?

Partly

Are sufficient details of methods and analysis provided to allow replication by others?

Partly

Reviewer Expertise:

Green/environmental marketing and Ethical Marketing.

I confirm that I have read this submission and believe that I have an appropriate level of expertise to confirm that it is of an acceptable scientific standard, however I have significant reservations, as outlined above.

# Response

**Published:** 2022-05-25

**Authors:** Jeen Wei Ong

**Affiliations:** Multimedia University , Malaysia

Dear reviewer,

 Thank you very much for your reviews and suggestions to improve the article. We have revised the article based on your suggestions. The amendments are summarised below.

 1. The literature review is part of the introduction section. More literature review is added to strengthen the argument on the existence of the research gap in terms of insufficient empirical evidence linking the green value chain to the firm performance.

 2. More information on the semi-structured interviews is provided, including verifying the capacity of the respondents to provide a valid response, the questions asked during the interviews and the process of converting the findings into the questionnaire item. 

 3. The research framework and hypotheses are added to the article.

 4. We take not on the inappropriate use of the term "worrying" in the article and have replaced the term with a neutral word.

 5. The implications of the study are moved to a separate paragraph and implications for policymakers are added. To comply with the format of the article, a separate section is not created.

 6. More discussion of the findings is added, especially in comparing the findings of our study with the previous studies and the suggestion to the policymakers.

# Peer review: Reviewer response for version 1

**DOI:** 10.5256/f1000research.77250.r126018

**Published:** 2022-03-24

**Authors:** Uchenna Cyril Eze

**Affiliations:** 1 Division of Business and Management, BNU-HKBU United International College, Zhuhai, China

The manuscript titled "The impact of green practices in the value chain on firm performance in the context of a developing country" appears well-written and provides insights in the domain of study, which although extremely important for industry operation, is rarely researched in the context presented. The objectives were clearly outlined and the findings appear valid. This manuscript, however, could have benefited from more clarity in the research method, more context in how the findings were presented, and some editing to remove obvious writing errors.

 There should be more details on how the semi-structured interview was conducted. For example, what were the positions of these managers for this reader to get a sense of the capability of the participants to provide reliable information? How much time elapsed between the interview and the survey, and how did this time affect the design of the survey questionnaire, if any?

 Provide more context in the discussion of the findings. How do the findings in this manuscript compare to the findings of prior studies in related area?

 Please check the overall manuscript to remove obvious grammar and typographical errors.

Is the work clearly and accurately presented and does it cite the current literature?

Yes

If applicable, is the statistical analysis and its interpretation appropriate?

Yes

Are all the source data underlying the results available to ensure full reproducibility?

Yes

Is the study design appropriate and is the work technically sound?

Yes

Are the conclusions drawn adequately supported by the results?

Yes

Are sufficient details of methods and analysis provided to allow replication by others?

Partly

Reviewer Expertise:

Mindful consumption, e-business, knowledge management, sustainability in business, green marketing, strategy

I confirm that I have read this submission and believe that I have an appropriate level of expertise to confirm that it is of an acceptable scientific standard, however I have significant reservations, as outlined above.

# Response

**Published:** 2022-05-25

**Authors:** Jeen Wei Ong

**Affiliations:** Multimedia University , Malaysia

Dear Reviewer,

 Thank you very much for your constructive review of this article. We have made the amendments as the following based on your suggestions.

 1. For your suggestion about the respondents, we added the description that the respondents are holding managerial positions in different business functions. We verify their capability to provide a reliable response by asking them to describe the company and their experience with the company.

 2. We also compare our results with the available results from previous studies. In general, our finding is consistent with the previous studies that green practices lead to greater performance. However, compared to the previous study that uses a dependent variable that comprises the triple bottom line, a discrepancy in the results is observed.

 3. We also recheck the article for obvious grammatical and typographical errors.
